# Reactive Oxygen Species, Apoptosis, Antimicrobial Peptides and Human Inflammatory Diseases

**DOI:** 10.3390/ph8020151

**Published:** 2015-04-02

**Authors:** Babatunji Emmanuel Oyinloye, Abiola Fatimah Adenowo, Abidemi Paul Kappo

**Affiliations:** 1 Biotechnology and Structural Biology (BSB) Group, Department of Biochemistry and Microbiology, University of Zululand, KwaDlangezwa 3886, South Africa; E-Mails: tunji4reele@yahoo.com (B.E.O.); afaatimah@yahoo.com (A.F.A.); 2 Department of Biochemistry, College of Sciences, Afe Babalola University, PMB 5454, Ado-Ekiti 360001, Nigeria

**Keywords:** antimicrobial peptides, apoptosis, inflammation, mitochondrial damage, nuclear factor kappa B, reactive oxygen species

## Abstract

Excessive free radical generation, especially reactive oxygen species (ROS) leading to oxidative stress in the biological system, has been implicated in the pathogenesis and pathological conditions associated with diverse human inflammatory diseases (HIDs). Although inflammation which is considered advantageous is a defensive mechanism in response to xenobiotics and foreign pathogen; as a result of cellular damage arising from oxidative stress, if uncontrolled, it may degenerate to chronic inflammation when the ROS levels exceed the antioxidant capacity. Therefore, in the normal resolution of inflammatory reactions, apoptosis is acknowledged to play a crucial role, while on the other hand, dysregulation in the induction of apoptosis by enhanced ROS production could also result in excessive apoptosis identified in the pathogenesis of HIDs. Apparently, a careful balance must be maintained in this complex environment. Antimicrobial peptides (AMPs) have been proposed in this review as an excellent candidate capable of playing prominent roles in maintaining this balance. Consequently, in novel drug design for the treatment and management of HIDs, AMPs are promising candidates owing to their size and multidimensional properties as well as their wide spectrum of activities and indications of reduced rate of resistance.

## 1. Introduction

ROS can be defined as oxygen-containing molecules, which are more reactive and potent than molecular oxygen itself [1,2]. ROS can be grouped into oxygen-centered radicals: hydroxyl radical (·OH), peroxyl radical (ROO·), alkoxyl radical (RO·), superoxide anion (·O_2_–) and oxygen-centered non-radicals: singlet oxygen (^1^O_2_) and hydrogen peroxide (H_2_O_2_) [3]. They are highly reactive molecules, which are generated during normal cellular metabolism or from external sources on exposure to chemicals, cigarette smoking, irradiation, air pollutants and ozone [4]. Sources of cellular ROS generation include phagosomes located in specialized cells of the immune system, involved in killing of pathogen; and peroxisomes, which facilitate catabolic oxidation reactions in favour of energy metabolism. Added to these, three other main sources involved in ROS formation within the cells are the endoplasmic reticulum, cell membranes and mitochondria [5]. When the level of ROS production exceeds the cellular antioxidants capacity, oxidative stress occurs [6].

Long term uncontrolled oxidative stress has been implicated in several human inflammation diseases (HIDs) such as hepatitis, asthma, cancer, vasospasms, stroke, retinal damage arthritis and atherosclerosis just to mention but a few [3]. This has placed HIDs at the forefront of the novel genetics revolution due to the significant rate of human morbidity of such diseases which are identified to be hereditary [7]. Free radicals, especially reactive oxygen species (ROS), have been identified to play a crucial role in the pathogenic processes in many of these diseases, ranging from responses to growth factor stimulation to generation of inflammatory responses. However, during normal metabolic processes, biological systems are continuously exposed to ROS, generated by different enzymatic and chemical reactions, which has been suggested to play essential roles in the regulation of physiological intracellular signal transductions, thereby acting as second messengers at low concentrations [8,9,10].

Growing evidence suggest that elevated levels of ROS result in an overwhelming effect on the endogenous neutralizing antioxidants, leading to increased oxidative stress and damage to proteins, carbohydrate, lipids, DNA and enzymes. It has been established that various downstream effects of increased oxidative stress (elevated ROS production) have a direct link with the induction of numerous inflammatory cascades [11,12]. In fact, recent studies showing interplay between ROS and inflammation, reports that ROS derived from mitochondria, directly stimulate molecules that incite the up-regulation of inflammatory cytokines through different molecular pathways [13,14].

Generally, inflammation is a normal protective response to a variety of cell and tissue damages, which is aimed at destroying and removing the detrimental and injurious cells and tissues, thereby initiating tissue repair. Uncontrolled inflammatory response results in extensive cell and tissue damages, giving rise to normal cell and tissue destruction, which is associated with chronic inflammation, implicated in various HIDs [10]. Mitochondria play a crucial role in cell survival, most importantly by generating adenosine triphosphate (ATP) as well as controlling apoptosis, cell cycle, and other essential metabolism. Through aerobic respiration, mitochondria generate ATP through oxidative phosphorylation; thereby glucose, pyruvate, and NADH are oxidized, thus generating ROS as a by-product. Interestingly, malfunctioning mitochondria have been implicated in various human diseases with underlying inflammatory pathologies [10].

Taken together, mitochondria and ROS play a prominent role under physiological and pathological conditions, during apoptosis induction. Apoptosis is an ordered cellular process triggered by various signalling pathways, notably, the intrinsic (or mitochondrial) and the extrinsic pathways; hence both pathways initiate caspases activation [15]. Central to the mechanism of apoptosis are caspases: the initiator caspases and executioner/effector caspases. The initiator caspases (caspase -8 and -9) activate the effector caspases (caspase -3, -6, and -7) in response to specific cell death signals. In the pathogenesis and progression of inflammatory diseases, due to continuous accumulation and activation of inflammatory cells, delayed apoptosis have been implicated [15]. To this end, for the resolution of inflammation, induction of apoptosis is also required [16]. Therefore, in the anticipation to gain novel insights into the constant inflammatory states that characterize inflammatory diseases, normal cellular processes of inflammation have been studied in relation to caspases activation.

Caspases are essential in maintaining homeostasis through cell regulatory networks; they are a family of endoproteases that provide significant links in controlling inflammation and apoptosis. They have been generally classified according to their identified roles e.g., in inflammation (caspase-1, -4, -5, and -12 are involved in humans while caspase-1, -11, and -12 in mice), while in apoptosis caspase -3, -6, -7, -8, and -9 are involved in mammals. A vast array of cellular stresses is known to activate the intrinsic apoptotic pathway [17]. In order to design and develop novel therapeutic agents with better efficacy in the treatment of HIDs, molecules capable of directly activating caspases while inducing apoptosis should be explored. Accumulating evidence suggest that antimicrobial peptides (AMPs) possess selective cytotoxic potential and induce apoptosis via intracellular ROS accumulation (oxidative stress) and mitochondrial damage [7,18,19,20,21].

Basically, treatment with AMPs promote the generation of ROS, even though ROS such as hydrogen peroxide and superoxide ion do not elicit crucial damage to cells per se, however, they may contribute in various apoptotic pathways as precursors of more potent ROS. For example, through electron leakage from respiratory chain in mitochondria, superoxide anion is produced from oxygen and this is converted to hydrogen peroxide (H_2_O_2_) by superoxide dismutase. Afterwards, H_2_O_2_ reacts with ferrous ion to produce highly reactive hydroxyl radical through the Fenton and Haber–Weiss reactions. Due to this, it is extremely easy for AMPs to induce plasma membrane disruption by directly damaging the plasma membrane or indirectly inducing apoptosis through intracellular ROS accumulation especially hydroxyl radicals and the release of cytochrome c via complete depolarization of mitochondrial membrane [19,20,21]. Therefore, antimicrobial AMPs may be a promising candidate capable of triggering apoptosis via caspase activation, due to their size and multifunctional properties.

## 2. Antimicrobial Peptides (AMPs): General Overview

Peptides possessing antimicrobial activities were initially described in 1966 by Zeya and Spitznagel [22]. Due to their varied functions, antimicrobial AMPs are otherwise referred to as multifunctional peptides [23,24]. For over three decades AMPs have been discovered in insects, amphibians, fish, birds, and mammals and they play a crucial role in the immunity of these creatures. AMPs are released by macrophages and granulocytes, several epithelial cells, small intestine (in the paneth cells), vaginal epithelium, oral cavity epithelium, airway epithelium, as well as skin glands of frogs [25]. Endogenous AMPs acts as natural antibiotics, they are constitutively expressed and in some cases induced swiftly to provide a quick and effective source of defence in response to pathogenic infections [26].

AMPs are mostly gene-encoded peptides with molecular weight of less than 10 kDa, usually positively charged, possessing amphipathic structure and binds to microbial cell wall or membrane through electrostatic and hydrophobic interactions thus causing membrane disruption [26]. Diverse AMPs have amino acid sequences, which are greatly varied, and their secondary structures are also different. Most AMPs have short length amino acid residues, ranging between 5 and 40, although a few contain over 40 residues [19]. Although, AMPs inhibit production of intracellular biomolecules, such as proteins, RNA and DNA without significantly permeating the cell membrane [26], yet, they are synthesized in a rapid and flexible manner. Due to their relatively small molecular size, AMPs are manufactured using a negligible input of energy and biomass [27]. In contrast to conventional antibiotics, AMPs usually act on quite a wide spectrum of microbes comprising parasites, enveloped viruses, fungi, gram negative and positive bacteria, as well as certain cancer cells [19].

Additionally, unlike conservative antibiotics, which generally aim at a specific metabolic enzyme and might induce resistance in microbes, AMPs kill microorganisms mostly by mechanism involving membrane disruption, which is naturally difficult for microorganisms to circumvent through development of resistance [19,28,29]. The spectra of antibiotic activity of individual peptide are dictated by the amino acid sequence as well as the structural conformation [23]. A significant attempt has been carried-out towards the development of certain classes of AMPs as pharmaceutical agents, generally for the management of external infections like oral mucositis-related infections, chronic infection of the lung connected with cystic fibrosis, diabetic ulcers, and ocular infections as well as bucco-dental infections; however none have attained clinical use [30].

## 3. Classification of AMPs

Antimicrobial peptides can be classified considering various factors such as origin of the peptide, the type of charge the peptide possess (positive or negative charges), amino acid content and the most recent classification is based on solution structure of peptides.

### Classification of AMPs Based on Structure

The solution structures of numerous peptides have lately been made available courtesy of Nuclear Magnetic Resonance (NMR) spectrometry. Structural classification is based on sequence homologies, functional similarities as well as three-dimensional structures. Based on this classification, AMPs can be categorised into five main groups. Firstly, linear α-helical peptides devoid of cysteine residue either with or without hinge region e.g., bombinins, cecropins and magainins. Secondly, AMPs having one disulfide bond which pack into a loop structure having a tail e.g., bactenecins and esculentins. Thirdly, AMPs having two or more disulfide bonds having mainly or just β-sheet structure such as defensins and protegrins. Fourthly, linear peptides devoid of cysteine residue and possessing a rare arrangement of regular amino acids like histatins, indolicidin and temporins. Lastly, AMPs resulting from larger proteins or peptides having other identified functions such as lactoferricins and MUC7 [25]. Table 1 shows some examples of antimicrobial peptides and their composition.

**Table 1:** Examples of AMPs and their composition (adapted from Oyinloye *et al.* [19]).

Antimicrobial peptides	Composition
Cecropins	Contains 31–39 amino acids with an amphipathic, basic N-terminal domain and a hydrophobic C-terminal domain.
Mellitin	Contains 26 amino acid residue peptide with distinct hydrophilic and hydrophobic domains
Maximins 1, 2, 3, 4 and 5	Contains 27 amino acid residues
Abaecin	Contains of 34 amino acids and contains almost 30% proline making it the largest proline rich antimicrobial peptide characterized, with broad spectrum of activity.
Magainin	Contains 23 amino acids residues
Hymenoptaecin	A glycine-rich antimicrobial peptide, containing 93 amino acids, with 2-pyrrolidone-5-carboxylic acid at the N-terminus.
Protegrins	Contains 16-18 amino-acid residues with four invariant cysteine residues, which form two disulfide bonds
Pleurocidin	Contains of 12 amino acid residue
Indolicidin	Composed of 13 amino acid residue containing five tryptophan and three proline residues
Bactenecin	Composed of 12 amino acid residue, including 4 arginine, 2 cysteine and 6 other hydrophobic residues

## 4. AMPs from Eukaryotes

Antimicrobial peptides from eukaryotic organisms can be broadly categorized into cationic peptides and anionic peptides.

### 4.1. Cationic Peptides

Cationic peptides make up the major group of peptides and are the first to be described, because they are broadly distributed among animals and plants. These include the following:

#### 4.1.1. Defensins

Defensins occurs naturally as antibiotic, cysteine-enriched cationic peptides. Different defensins have so far been isolated from plants, mammals and insects. They exhibit significant antibacterial, antifungal as well as antiviral action and function as molecules that ensure intrinsic immunity [30,31]. Three defensins have been discovered in humans namely: human neutrophils peptides-1 to -3 (HNP-1 to -3); they make-up over 5% of the entire protein in human neutrophils. These defensins (HNP-1 to -3) molecules encompass a hydrophobic area that is attached in the lipid part of membranes; they are integrated into prokaryotes cell membrane throughout phagocytosis, disturbing ion stabilities, thus effecting lysis of the cell. The concentration of defensins within the plasma is usually lower than 15 nM, nonetheless concentrations up to 50 µM sometimes occur in patients having sepsis and bacterial meningitis [32]. Presently, six α-defensins and four β-defensins (hBD1–4) have been well characterized [33]. In humans, epithelial cells of several organs produce beta-defensins. Generally, β-defensins display a broad spectrum of antimicrobial activity. Although, the family of human β-defensins (hBD) consist of at least 30 members; to date, subfamily members of hBD-1 and hBD-2 are the most studied while hBD-3 and hBD-4 are less studied [34]. Upon stimulation by diverse inflammatory factors, they are either constitutively expressed or induced. In a concentration-dependent manner, hBD-4 exerts its biological actives and may be potentially used for wound healing or as anticancer agents [35].

Plant defensins usually comprises of about 45 to 54 amino acids residues, they possess a net positive charge, and display clear, though comparatively limited sequence conservation. The amino acid residues conserved in every sequence are limited to the eight cysteine and two glycine residues at positions 13 and 34 respectively, an aromatic amino acid residue on position 11, and a glutamine at the 29th position [27]. Majority of plant defensins presently isolated are derived from seeds, many have been subsequently characterized at the biochemical, molecular as well as at the structural levels [36,37,38]. In contrast to the mammalian and insect defensins, which are mostly active against bacteria, plant defensins, although with limited exemptions do not exhibit antibacterial action. Majority of plant-derived peptides are intricate in defence against a wide variety of fungi. Plant defensins are not just potent against phytopathogenic fungi like *Botrytis cinerea* and *Fusarium culmorum* but are also potent against human pathogenic fungi like *Candida albicans* and baker’s yeast [36,38].

Insect defensins are very potent against Gram-positive microorganisms like *Staphylococcus aureus*, however they present weak activity against Gram-negative bacteria. They have been isolated from numerous insect orders like diptera, hymenoptera, hemiptera, trichoptera, coleoptera and odonata [39].

#### 4.1.2. Cathelicidins

Cathelicidins consist of collection of mammalian-derived cationic antimicrobial peptides having a shared genetic basis but presenting a significant diversity of sequences, sizes as well as structures [40]. Altogether, cathelicidins comprise an N-terminal cathelin domain and a C-terminal cationic antimicrobial domain, which is activated upon cleavage. Similar to defensins, these peptides are vital constituents of immune defence. Added to their different antimicrobial activities, cathelicidins have the ability to bind to endotoxins and neutralize their effects, thus they are excellent candidates for therapeutic applications. They are amphipathic molecules that inhibit microbial functionality by targeting the membranes. More so, cathelicidins cooperate with human host recognition receptors to trigger cellular immune defence [25].

Over 30 cathelicidins have so far been discovered in mammals; nonetheless, just acathelicidin (LL-37) has been discovered in humans [41,42]. LL-37 is abundant in the human body; it has been studied to show a wide range of antimicrobial action against a diversity of bacteria, fungi as well as virus. It plays vital role in healing of impaired tissue as well as wound healing, through supporting re-epithelialization of restorative skin and wound neovascularization [43].

#### 4.1.3. Cecropins

Cecropins are linear amphipathic peptides about 3–4 kDa that show activity against bacteria, fungi, protozoa as well as metazoan parasites, belonging to the family of antibacterial peptides having 35–37 amino acids residues. It was originally discovered in cecropia moth (*Hyalophora cecropia*) however, other peptides derived from other insect species as well as mammals are also included in the family [26]. Cecropins lacks cysteines but have dual characteristic helical segments, a very basic N-terminal domain as well as an elongated hydrophobic C-terminal helix, which is connected by a small hinge. When combined with melittin, cecropins are promising anticancer drugs [44].

#### 4.1.4. Thionins

Thionins are set of small cysteine-rich and very basic peptides approximately 5 kDa, which are understood to play a vital role in the fortification of plants against infection by microbes. They are present in the stems, seed endosperm, roots, as well as leaves of pathogen-stressed plant species. Thionins are lethal to Gram-positive and Gram-negative bacteria, yeasts, fungi, as well as numerous mammalian cell types. Toxic effect of thionins needs an electrostatic interface of the peptide with membrane phospholipids that are negatively charged, followed by pore formation or interaction with a specific domain in the membrane [45]. Thionins are divided into minimum of four diverse types depending on the plant species and tissue where they are present in, the number of amino acid residues as well as number of disulphide bonds and the overall charge [46]. A good example of thionins is Ligatoxin B isolated from the mistletoe, *Phoradendron liga*, comprising of 46 amino acid residues [47].

#### 4.1.5. Amino Acid-Enriched Antimicrobial Peptides

Amino acid-enriched AMPs are distinctive antifungal and antibacterial cationic peptides, enhanced with definite amino acids; they have unique structures depending on the organism they are derived from. Glycine- as well as proline-rich peptides are typically isolated from insects; they are potent on Gram-negative microbes [47,48]; whereas cysteine-enriched peptides, which are not associated to defensins, symbolise a different family amongst arthropods. However, those peptides rich in histidine are predominantly basic and typically of mammalian origin [47,49,50]. Amongst them, the peptide histatin isolated from saliva of primates and humans which is effective against fungal pathogens, is outstanding for its unique mode of action that does not include formation of channel in the cytoplasmic membrane of fungi but somewhat translocate proficiently inside the cell, targeting the mitochondrion [51].

#### 4.1.6. Histone Derived Peptides

This family of cationic peptides, which is analogous to cleaved forms of histones was firstly isolated from toad (called buforin) and the epithelia fish (referred to as parasin) [52,53]. These peptides are structurally related to cecropins and are quite potent against fungi and bacteria. Buforin II was demonstrated to infiltrate bacterial membranes binding to nucleic acids hence disrupting cell metabolism and causing rapid cell death [47,53].

### 4.2. Anionic Peptides

Anionic peptides are small group of peptides with antimicrobial activity; these peptides have been mainly isolated from mammals. Members of the group include:

### 4.3. Neuropeptide Derived Molecules

Neuropeptides were discovered from infectious fluids of humans and cattle. They commonly comprise peptides resulting from the processing of neuropeptide precursors like pro-enkephalin-A, to give active peptide B and enkelytin; many of which are phosphorylated. They are chiefly potent against Gram-positive pathogens at micromolar concentrations [54,55].

### 4.4. Aspartic-Acid-Rich Peptides

These peptides were discovered and subsequently characterized mainly from the pulmonary fluids of cattle [56,57]. Their structure is analogous to the charge-neutralizing pro-peptides belonging to Group I serine proteases; they were suggested to control the action of pulmonary enzyme of cattle. In recent times, a new 47-amino-acid anionic peptide called dermicidin was discovered in human exudate, and in reaction to a diversity of Gram-positive bacteria, this peptide was also classed into this group [58].

## 5. Antimicrobial Peptides from Prokaryotes

AMPs produced by bacteria are grouped into various classes on the basis of the producer organisms, chemical structure, molecular size, and mode of action, thus resulting into diverse names for recognised peptides that are identical namely: bacteriocin, thiolbiotics, colicin, lantibiotic and microcin amongst others [59]. The most pertinent amongst them are derived from gram-positive bacteria and named bacteriocins, while the most comprehensively studied are prokaryotes-derived peptides released by lactic-acid bacteria, lantibiotics, which is made up of modified amino acid residues [47,60].

## 6. Role of ROS in Human Inflammatory Diseases (HIDS)

In recent years, ROS has been implicated in several biological activities, spanning a wide spectrum, ranging from physiologic regulatory processes to pathogenic conditions identified in the pathogenesis of various diseases [9,10,11]. As a matter of fact, uncontrolled and excessive generation of ROS (results in oxidative stress), especially mitochondria-derived ROS (mtROS) directly stimulate up-regulation of inflammatory cytokines associated with diverse pathological conditions in HIDs [15]. In addition, inflammation occurring as a result of oxidative stress accounts for a number of HIDs; yet, inflammation is not the only biological manifestation of uncontrolled and excessive generation of ROS. Nevertheless, in response to free radical overload that commonly arise from mitochondria; a cascade of pathological inflammation, a complex cellular pathway begins and activate several signaling molecules [61].

One of the key mediator signaling molecules activated is the transcription factor nuclear factor kappa-B (NF-*κ*B), that up-regulates the production of downstream inflammatory mediators such as inducible NO synthase (iNOS), interleukin-1β (IL-1β), tumor necrosis factor-α (TNF-α) and cyclooxygenase-2 (COX-2). NF-*κ*B plays significant roles in inflammation and apoptosis, immune and stress responses as well as regulation of expression of various genes [61,62]. Uncontrolled and excessive generation of ROS therefore, is capable of causing oxidative modification and damage to cellular bio-molecules such as lipids, carbohydrates, proteins and DNA via different mechanisms by impairing their functions permanently. One of such is formation of lipid peroxidation and lipid-derived aldehydes, which participates in various free radical reactions. Lipid peroxidation products and lipid-derived aldehydes are highly deleterious, and competent of compromising the membrane integrity resulting in loss of cellular functions and ultimately severe cytotoxicity manifested in various HIDs [63].

Lipid peroxidation products and lipid-derived aldehydes such as malondialdehyde (MDA), 4-hydroxy-2-nonenal (HNE) and acrolein have been implicated in numerous oxidative stress-induced HIDs [63]. Proteins and DNA may also be damaged by ROS, resulting in various structural modifications and impaired enzyme activities as well as formation of various oxidative DNA lesions which is capable of inducing mutations and loss of cell viability [62,64]. Among the oxygen-based radicals, hydroxyl radical is the most toxic eliciting its deleterious effects within cells, mainly with macromolecules [64]. Although the body has numerous mechanisms in place to neutralize these deleterious effects, (DNA repair enzymes and/or antioxidants), growing evidence of excessive free radicals generation, especially ROS leading to oxidative stress in the etiology of a number of chronic and degenerative diseases including HIDs is overwhelming [62].

**Figure 1:**
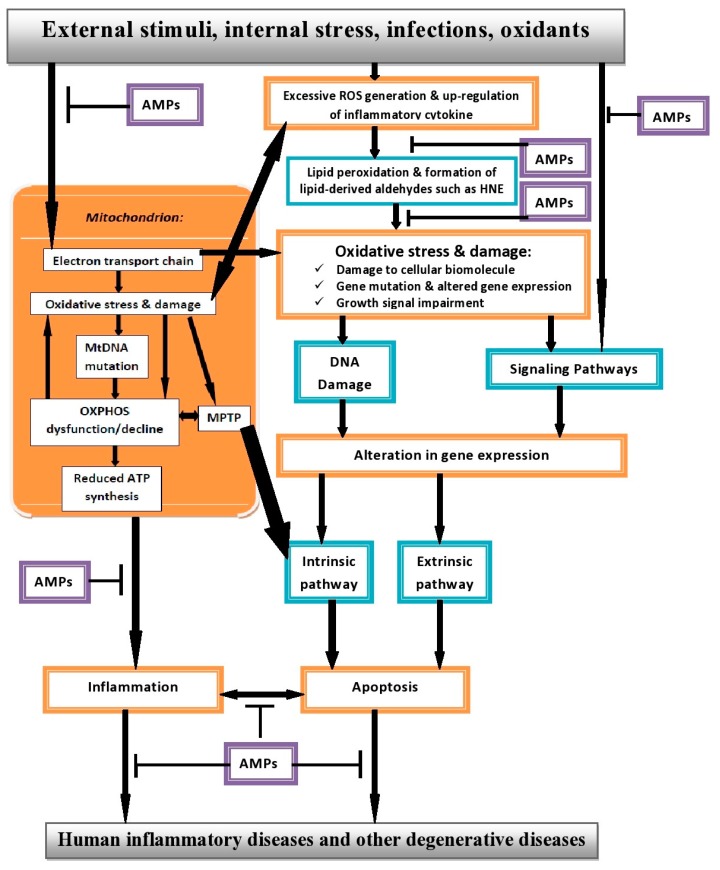
Proposed model of interaction between ROS, apoptosis and AMPs in HIDs.

Taken together, inflammation develops as one of the biochemical response to the deleterious effects of ROS on biological systems and eventually degenerates to various forms of HIDs, particularly when not adequately treated, due to cellular stress and dysfunction arising from severe damages and alterations to cellular biomolecules. Figure 1 shows the proposed model of action of excessive ROS generation leading to diverse HIDs.

## 7. Impact of Apoptosis in Human Inflammatory Diseases (HIDs)

Clear evidence indicating that regulated apoptosis plays a vital role in preventing normal inflammatory response from degenerating to chronic inflammation as well as the diverse pathologic conditions associated with HIDs has emerge [65]. Inflammatory response has been identified as an imperative and efficient system of host defence against chemical insult, infection and injury. Although, initially it was thought to be an entirely beneficial process; but in recent years, growing evidence has revealed the significant roles played by inflammation in the pathogenesis of a number of diseases such as hepatitis, asthma, myocardial infarction, Alzheimer, glomerulonephritides, Parkinson amongst others [65]. Continuous accumulation and activation of inflammatory cells is connected with disruption of normal tissue architectures and tissue injury as well as excessive fibro-proliferative responses that lead to impairment of organs and failure in many HIDs [65].

Alteration in tissue homeostasis has significant implications in pathology. Apoptosis, a basic regulated biological process essential for development and regulation of tissue homeostasis occurs as a result of caspase activation. Two key molecular pathways facilitate the activation and consequent accomplishment of apoptosis [66]. One is the extrinsic/death receptor pathway which is activated in response to extracellular signals/initiated by ligand-induced accumulation of death receptors like Fas and tumor necrosis factors, while the other is the intrinsic or the mitochondria-dependent pathway; triggered in response to a number of stress conditions especially oxidative stress, causing DNA damage. Basically, the overall stress effect is directed towards the mitochondria, this in turn affects ATP production. Consequently, a number of proteins are released that contribute to caspase activation in the intrinsic apoptotic pathway. Alteration in this vital regulated process results in either too little or too much apoptosis; hence this has been implicated in the pathogenesis of various HIDs [66,67,68,69].

## 8. AMPs with Apoptotic and Cytotoxicity Activities

As mentioned above, apoptosis is a vital process and a distinctive type of cell death, which performs essential role in the development of embryo, development of the immune system and carcinogenesis. Generally, the characteristics of apoptotic cells include cell shrinkage, chromatin condensation, DNA fragmentation, cell surface expression of phosphatidylserine, as well as membrane blebbing [68]. Aerts and co-workers show that *Raphanus sativus* antifungal protein 2 (RsAFP2) an antifungal plant defensin isolated from radish-triggered apoptosis and concurrently initiates the action of caspases in the pathogenic organism, *C. albicans* present in human. In addition, they establish that removal of *C. albicans* metacaspase 1, which encode the only described (putative) caspase in *C. albicans*, considerably affects caspase activation. They conclude that the study offers the first proof of an antifungal antimicrobial peptide, which induces apoptosis in *C. albicans*. Furthermore, the study indicate potential medical application of plant defensins particularly RsAFP2 in the management of *C. albicans* infections [69].

Cho and Lee suggested that arenicin-1, a twenty-one residue AMP displays antifungal activity by inducing apoptosis in *C. albicans* through intracellular reactive oxygen accumulation, triggering the depolarization of the mitochondrial membrane and the release of activated metacaspases. It also initiated plasma membrane depolarization and the release of phosphatidylserine on the membrane surface. The cell that experiences the aforementioned phenomenon exhibits morphological alterations in the nucleus as well as DNA structural changes and hence cell death [20].

Lee and co-workers studied the induction of apoptosis by coprisin in *C. albicans* cells. Coprisin, a defensin-like peptide isolated from the dung beetle *Copris tripartitus* exerted fungicidal activity devoid of any haemolytic effect. TUNEL assay and Annexin V-FITC staining established that coprisin was intricate in both early as well as late stages of apoptosis. Coprisin increased the intracellular levels of ROS as well as hydroxyl radicals. It also induces potential dysfunctioning of mitochondrial membrane, release of cytochrome C and metacaspases activation [70].

Cecropin A, a straight chain 37-residue AMP produced by cecropia moth has demonstrated cytotoxic effect on different human cancer cell lines as well as inhibitory action on growth of tumour. This AMP was studied to induce apoptosis in human promyelocytic cell line. As a result of treating cell line with cecropin A, a dose-dependent loss of cell viability, leakage of lactate dehydrogenase (LDH), increase in ROS generation, attenuation of lysosomal integrity as well as fragmentation of DNA, externalization of phophatidylserine and nuclear condensation occur. However, the induction of apoptosis by cercropin was not dependent on caspases [71].

## 9. AMPs and Human Inflammatory Diseases

Present and future researches are expected to explore the potentials of antimicrobial peptides in order to offer novel therapeutic lines for the treatment and management of various HIDs [72]. The following are some human inflammatory diseases and research outcomes on the role of AMPs in the management of the diseases.

### 9.1. Atherosclerosis

Development and progression of atherosclerosis an inflammatory disease, begins during foetal stage of development and advances over periods under the impact of genetic as well as environmental factors. In spite of modifications in way of life and the usage of novel pharmacological agents, it remains one of the chief sources of mortality in Europe, United States, and a considerable percentage of Asia, causing an enormous social and financial encumbrance in contemporary societies. Numerous predisposing factors like diabetes, hyperlipidemia, hypertension or smoking were recognised, however these factors were projected to represent merely partial aspect of the individual variable risk of having atherosclerosis [31]. Retaining of low-density lipoproteins inside the walls of blood vessels as well as the response of endothelial cell to a wide variety of harmful stimuli are amongst the general theories, which try to unite experimental facts along with the observed clinical development of the syndrome. Today, it is generally acknowledged that the incidence of failure in endothelium function irrespective of disease origin is a vital occurrence which starts-off and proliferates atherosclerotic progression [73].

Edfeldt and co-workers investigated the role of a human cathelicidin AMP, LL-37 in the process of atherosclerosis. By means of real-time polymerase chain reaction (RT-PCR), a 6-fold rise in human cationic LL-37 transcript in atherosclerotic lesions in comparison with normal arteries was discovered. More so, immunohistochemical analysis of plaques from atherosclerotic patients indicates that peptide LL-37 was induced mostly by macrophages and certain endothelial cells. Western blot analysis confirmed the presence of active LL-37 peptide in atheroma specimens. To comprehend the functional effect of LL-37 expression in atherosclerosis, the transcription profile was evaluated in endothelial cells administered with LL-37. The result indicated LL-37 is expressed in atherosclerotic lesions, wherein it might function as an immunomodulatory agent through the activation of adhesion molecules and expression of chemokines, hence improving innate immunity in atherosclerotic patients [74].

Similarly, Ciornei and co-workers studied the occurrence of LL-37 peptide in atherosclerotic lesions of humans acquired at post-mortem through immunohistochemistry. They studied the outcome of the peptide on isolated neutrophil granulocytes and cultured vascular smooth muscle cells through morphological, biochemical as well as flow cytometry analysis. In smooth muscle cells cultures, LL-37 at a concentration of 30 µg/mL produced nuclear condensation, cell shrinkage, membrane blebbing, fragmentation of DNA as well as an upsurge in caspase-3 action as revealed by cell microscopy, ELISA and enzyme activity assay. These results established that LL-37 exists in lesions due to atherosclerosis and prompts loss of cultured vascular smooth muscle cells, mostly through an influence on the permeability of the plasma membrane thus, causing cell death. Conclusively, the results propose a function for LL-37 in inducing apoptosis in atherosclerotic lesions [75].

### 9.2. Cancer

In spite of current improvements in management modalities, cancer persists as chief source of ill health and death globally. Cancer is a foremost cause of death for people lower than 85 years of age in the United States. Furthermore, the prevalence of numerous cancers, such as cancers of the kidney, skin, breast, and prostate is on the increase [76]. Cancer refers to a general term, which denotes over hundred diverse diseases afflicting different tissues as well as cell types. Nevertheless, all classes of cancer are branded by abnormal growth of cell ensuing from a comparatively few number of genetically inherited mutation or those that are induced environmentally [77]. Some AMPs are exclusively auspicious candidates vital for cancer treatment in humans due to numerous unique features. Firstly, their selective activities on malignant cells as well as their noticeable lytic actions on high-quality tumour cells, permit for an optimum treatment *in vivo* with little therapeutic concentrations as well as minimal side effects.

Secondly, AMPs have been established to have substantial lytic action against multi-drug resistant cancer cells, and lastly AMP anticancer agents are resistant against urine and serum proteolysis due to their biochemical structures, thus making them perfect candidates for the management of intravesical tumor [78]. However, not all antimicrobial peptides are capable of killing cancer cells, AMPs with anticancer activity can be classified into two general categories namely: AMPs which are extremely effective against cancer cells and bacteria but not potent against normal cells of mammals and those that are cytotoxic against bacteria, cancer cells, as well as normal cells of mammals [76]. Suttmann and co-workers studied the antitumor efficacy of two members of the cecropins family cecropins A and B on bladder tumour cells and evaluated their prospective activity as a novel treatment alternative for intravesical management of non-muscle invasive bladder cancer. Both peptides exerted selective cytotoxic as well as antiproliferative ability in bladder tumour cells in a dose-dependent manner while human fibroblast cells were not affected. Hence, both peptides might proffer new therapeutic approaches for the management of cancer of the bladder while exhibiting restricted cytotoxic action upon non-malignant cells [78].

Ohsaki and co-workers demonstrated the antitumor activity of two analogues of cationic AMPs magainin; magainin A and magainin G. They reported that the two peptides exhibited comparably consistent dose-dependent anticancer activity against six small cell lung cancer cell lines. Conversely, these peptides had less effect against benign human fibroblast cells than cancerous cell. Both magainin analogues exhibited constant tumour growth inhibition on the six lung cancer cell lines having a 50% inhibitory concentration (IC_50_) values in a 3-fold range [79]. They also assessed the effect of combination of magainin analogues with two chemotherapeutic agents; DDP and VP-16. Both analogues demonstrated an added antitumor effect in combination with standard drugs. The mechanism behind the antitumor action of magainin analogues is yet to be fully elucidated, however, the development of voltage-dependent ion channels that modifies membrane potential and functionality is considered to be a likely mechanism of cytotoxic and antimicrobial activity of magainin peptides [79].

Baker and co-workers assessed natural magainins and two magainin analogues for their *in vivo* anticancer activity. They designed the magainin analogue sequence to improve the amphiphilic α-helical structure and to reduce the vulnerability to proteolytic degradation. They demonstrated that natural magainin 2 and its synthesized analogue MSI-238 and MSI-136 has *in vivo* anticancer ability against four murine peritoneal ascites tumours; two murine leukemias cells (L1210 and P388), an ascite sarcoma cell (S180) as well as a murine ovarian teratoma (SOT). MSI-238 showed more effect than MSI-136 while both showed more cytotoxicity than the natural compound magainin 2 [80].

### 9.3. Helicobacter Pylori Infections

Over two decades ago, Barry Marshall and Robin Warren reported the discovery and culturing of a spiral-shaped bacterial species, subsequently referred to as *Helicobacter pylori* from the human stomach. Self-ingestion experimentations done by Marshall and later researched with volunteers established that these species of bacteria are capable of colonizing the human stomach, thus inducing inflammation of the gastric mucosa [81]. *H. pylori* septicity is a significant public health challenge in numerous countries of the world. Since this bacteria is responsible for many gastric diseases, including superficial gastritis, chronic atrophic gastritis, peptic and duodenal ulcer and gastric cancer, considerable attention is vested on how infection due to *H. pylori* can be prevented [82]. This pathogen has virulence elements, which are essential to colonise stomach acid milieu and thus ensure its survival; these factors are present in all isolates. The most significant factors are adhesins and urease. Urease ensures metabolism of urea to ammonia as well as carbon dioxide, and also assist in neutralising gastric acid. More so, urease is intensely immunogenic for phagocytes, promoting the synthesis of pro-inflammatory cytokines interleukin IL-6 and IL-8, (IL)-1β and tumor necrosis factor-α (TNF-α) [83].

*H. pylori* adheres precisely to the gastric mucosa epithelium cells courtesy of adhesins; mostly studied adhesins include Sab A and Bab A, these are outer membrane proteins linked to antigens of blood group Lewis-x and Lewis-b respectively [84,85]. *H. pylori* neutrophil-activating protein (HP-NAP) permits the pathogen to absorb iron needed for its activity. HP-NAP is predominantly vital in the mechanism of development of the *H. pylori* infection since it stimulates adhesion as well as chemotaxis of phagocytes [84,85]. HP-NAP stimulates the NADPH oxidase enzyme that is intricate in the release of reactive species of oxygen (ROS). HP-NAP also activates the release of cytokines (IL-12 and IL-23) via neutrophils and monocytes; IL-12 and IL-23 promotes inflammation. Due to its characteristics, HP-NAP leads to proliferation of gastric mucosa inflammation, thus the incessant harm to gastrointestinal cells instigated by ROS [84,86,87].

Iwahori and co-workers investigated the efficacy of normal magainin 2 and its synthetic analogues against two strains of *H. pylori*, ATCC 43526 and ATCC 43579. One of the analogues, MSI-78A exhibited the best activity against the bacteria with a minimum inhibitory concentration (MIC) of 8. Biophysical analysis of the peptide using circular dichroism (CD) indicated that the most active peptide (MSI-78A) had a greater α-content. They also discovered that five positively charged amino acids KILKK on the c-terminal of the amphipatic α-helix structure perform crucial roles in the peptide activity on *H. pylori* [88]. The aforementioned research group later synthesized (±) 6-benzyl-1-(3-carboxylpropyl) indane (PM2c) from magainin 2 and tested its ability to impede the growth of strains of *H. pylori*. Their result showed the synthesized peptide might be very valuable alone or in conjunction with conventional therapeutic agents for the management of *H. pylori* infection [89].

Furthermore, an anti-adhesive peptide potent against *H. pylori* infection was obtained from the enzymatic hydrolysis of proteins from the seeds of *Pisum sativum* (Pea). MALDI-TOF-MS was used to identify bioactive anti-adhesive peptide S3 and S5. These peptides inhibited the bacterial adhesion Bab A; an outer membrane protein needed for the adhesion of *H. pylori* to human gastric epithelial cells. It was concluded that bioactive peptides isolated from *P. sativum* protein might be useful as ingredients for protection against *H. pylori* [90].

Bajaj-Elliott and co-workers assessed the role of β-defensin in the immune response of the gastrointestinal epithelium to infection by evaluating mRNA expression and regulation of human beta-defensins 1 and 2 (hBD1 and hBD2) by *H. pylori* in three gastric epithelial cell lines; MKN45, MKN7 and AGS using quantitative reverse transcription polymerase chain reaction (RT-PCR). They discovered that cytotoxic *H. pylori* significantly up-regulated the expression of hBD2 and hBD1 in a dose as well as time dependent manner in the MKN7 and AGS cell lines, however the rate of mRNA expression was considerably slower in hBD1. *In vivo* studies were also carried out to observe the effect of *H. pylori* on hBD1 and hBD2 mRNA expression in gastrointestinal mucosa. RT-PCR performed on biopsies from *H. pylori* positive patients indicated considerable amount of hBD2 while the control tissue of patients without infection showed the absence of hBD2. These findings indicated the relevance of beta-defensins in *H. pylori* infection [91].

In another study, Rigano and co-workers used bioinformatics to identify putative defensins in the genome of *Lycopersicum esculentus* (tomato). The group chemically synthesized the γ-motif of tomato defensin and tested its antimicrobial potency against Gram-positive and Gram-negative bacteria including *H. pylori* cell lines (THP-1). The peptide exerted an anti-inflammatory action *in vitro*; it down-regulated the level of pro-inflammatory cytokine (IFN- γ) and tumor necrosis factor-α (TNF-α) [92].

### 9.4. Cystic Fibrosis

Cystic fibrosis (CF) was originally acknowledged as a distinct syndrome in 1938 after post-mortem examination of undernourished children differentiated a disease of phlegm blocking of the glandular vessels, named “cystic fibrosis of the pancreas,” from other children having celiac syndrome. Cystic fibrosis (CF) was depicted by malabsorption of protein and fat, growth failure, as well as pulmonary infection. Impairment of the pancreas and deficiency of pancreatic enzyme exudation is responsible for nutritional let-down, causing susceptibility to lung infection, which is frequently a life-threatening event. The presence of thick, sultry phlegm blocking the tubes of salivary glands led to the alternative name “mucoviscidosis” [93].

CF is a heterogeneous recessive genetic disorder having characteristics that reveal mutations in the cystic fibrosis transmembrane conductance regulator (*CFTR*) gene. Classic cystic fibrosis replicates two malfunctioning transmutations in the *CFTR* gene and is represented by protracted bacterial infection of the sinuses and airways, fat maldigestion owing to pancreatic exocrine inadequacy, infertility in men due to disruptive azoospermia, as well as high concentrations of chloride in perspiration. Patients having non-classic cystic fibrosis possess a minimum of a replica of a mutant gene which gives incomplete functioning of the *CFTR* gene, and the patients generally have no evident symptoms of improper digestion since some pancreatic exocrine functioning is preserved [94].

Health workers handling individuals with CF are progressively confronted with infections instigated by multidrug-resistant strains. However, *Pseudomonas aeruginosa*, *Stenotrophomonas maltophilia* as well as *Staphylococcus aureus* remains the commonest bacteria isolated from patients airways wherein they instigates insistent infections connected with a further speedy degeneration in lung functioning as well as survival [95,96]. More so, attempts to cure CF infections are hindered by the great bacterial adaption to the CF pulmonary milieu, causing a better capability to form biofilms naturally resistant to conventional antibiotics such as tetracycline, aminoglycosides and fluoroquinolones. Hence, new antimicrobial agents, which can substitute or complement existing therapies are thus desirable to combat prolonged infections in CF patients [97,98,99,100].

Pompilio and colleagues studied *in vitro* antibacterial and anti-biofilm activity of two bovine α-helical AMPs; BMAP-27 and BMAP-28 and an artificial peptide; P19(9/B) against twenty-five *Pseudomonas aeruginosa*, fifteen *Staphylococcus aureus* and twenty-seven *Stenotrophomonas maltophilia* strains isolated from cystic fibrosis patients. They compared the peptides efficacy with Tobramycin, the drug for the inhalation treatment of protracted airway infections in cystic fibrosis patients. Assessment of minimum inhibitory concentration (MIC) and minimum bactericidal concentrations (MBCs) values, as well as time killing assays against isolated CF pathogens revealed that the three peptides are very active against almost all the studied strains; with BMAP-28 exhibiting the broadest spectrum of action. However, it was notable that all tested peptides demonstrated higher potency than the standard antibiotic Tobramycin. The results also indicated that all tested peptides showed ability to decrease biofilm formation, though largely at a lesser degree than Tobramycin [96].

In order to identify therapeutic agent for cystic fibrosis lung, Zhang and co-workers investigated 150 antimicrobial peptides comprising three different structural classes and screened them against multi-drug resistant isolates of *Pseudomonas aeruginosa*, *Stenotrophomonas maltophilia*, *Staphylococcus aureus* and *Achromobacter xylosoxidans*. The lead peptides were selected on basis of optimal MICs against aforementioned clinical isolates, which are involved in CF lung pathogenesis. The reported *in vitro* antibacterial analysis of many of the peptides was greater than that of many conventional antibiotics. Added to bactericidal activity against multiple microorganisms, certain peptides also have activity against Gram-positive bacteria and *C. albicans*. This is an added advantage because those pathogens could be present in the lungs of CF patients and other conventional antibiotics for CF therapy, like tobramycin usually lack worthwhile gram-positive as well as fungal coverage. Thereafter, five peptides, which demonstrated strong antimicrobial actions in physiological salt as well as divalent cation medium, were characterized *in vivo* with rat model with chronic lung infection. The animal studies with these peptides showed better effectiveness in fast-tracking bacterial elimination from the lungs of infected rats [100].

Lawyer and colleagues produced a C-terminal portion of tracheal peptide (TAP) having 38 amino acids, thereafter; they studied it for effectiveness against several isolates of *P. aeruginosa* strains from CF patients and *Aspergillus fumigatus*. Their results showed that TAP possesses both potent bactericidal as well as fungicidal activities. More so, they demonstrated that a combination of TAP and the standard drug amphotericin B exhibited high additive action of growth inhibition on *Aspergillus fumigatus*. The results indicated that TAP could be a potential effective treatment for *Pseudomonas* and *Aspergillus* in cystic fibrosis patients [101].

## 10. Interplay between ROS, Apoptosis and AMPs in Human Inflammatory Diseases (HIDs)

Although inflammation, which is a defensive mechanism in response to xenobiotics and foreign pathogens as a result of cellular damage is considered advantageous, but if uncontrolled, may degenerate to chronic inflammation [102,103,104]. Biological systems are known to generate ROS under normal physiological conditions. Even though ROS at low concentration have been established to play a prominent role in a number of regulatory processes, the exact concentration is still undefined [9,10,11,104]. However, biological systems have several mechanisms in place, through the antioxidant defence system that neutralizes and prevents cellular damage as a result of ROS generation. When ROS levels exceed the cellular antioxidant capacity, it results in oxidative stress, which enhances the inflammatory reaction, a state identified to be central in the pathogenesis and progression of many HIDs [104].

Owing to the fact that enhanced production of ROS results in various cellular alterations and damages which favours inflammatory reactions and may lead to chronic inflammation, ROS at higher concentration also triggers the intrinsic apoptotic pathway leading to caspase activation and the consequent opening of the mitochondrial membrane permeability pore (MMPP) forcing the release of cytochrome c [105]; which plays a vital role during inflammation and in normal inflammatory reactions because central to the mechanism of apoptosis, is enhanced production of ROS and caspase activation [104,105].

This can be viewed as a two-edged sword because enhanced ROS production leads to the induction of apoptosis in normal inflammatory reactions, which is advantageous to the system. Conversely, dysregulation of this same mechanism of inflammatory reactions through induction of apoptosis by enhance ROS production could also result in excessive apoptosis identified in the pathogenesis of HIDs [66,106]. Therefore, a careful balance must be maintained, preventing inflammatory reactions from resulting in excessive apoptosis. Apparently, AMPs could play a prominent role in maintaining balance in this complex environment because of their multidimensional properties capable of resolving inflammatory processes, inhibits apoptosis as well as inducing apoptosis during inflammatory process as at when due via different mechanisms ensuring a tightly coordinated and regulated process [107,108,109,110,111].

Recent scientific advances as touching the immunomodulatory properties of AMPs especially beta-defensins and cathelicidins (LL-37) has been documented. AMPs could then be considered to possess both pro-inflammatory and anti-inflammatory properties, suggesting that they are key players in the inflammatory microenvironment [112,113,114]. For instance, Schuerholz and co-workers reported that hBD3 downregulates pro-inflammatory cytokines like TNF-α or IL-6 in human and mouse macrophages after exposure to LPS *in vitro* and *in vivo* [109]. More so, Campbell and co-workers reported that hBD3 upregulates COX-2 and PGE2 biosynthesis in gingival fibroblasts. hBD3 antagonize T-cell tissue infiltration and promote exfiltration [113] thus, suggesting the role of hBD3 in the resolution of inflammatory processes. On the other hand, LL-37 stimulates the release of anti-inflammatory PGD2 from mast cells, which can prime tissues by expressing enzymes necessary for resolution [112].

Aside resolving inflammatory processes, AMPs are also capable of inhibiting apoptosis. Ramanathan and co-workers reported that among the cathelicidins, porcine PR-39, a proline–arginine rich 39 amino acid, have been identified to inhibit apoptosis induced by a variety of stimuli. They concluded that this effect was associated with lower caspase-3 activity [108]. Added to this, Nagaoka and co-workers investigated the action of hBD-1 to -4 on neutrophil apoptosis using human blood neutrophils based on their morphological changes. Among hBD-1 to -4, hBD-3 most potently suppressed neutrophil apoptosis, which was associated with down-regulation of truncated Bid (a pro-apoptotic protein), up-regulation of Bcl-x_L_ (an anti-apoptotic protein) and inhibition of mitochondrial membrane potential change and caspase-3 activity. They concluded that hBDs, especially hBD-3, can not only kill bacteria but also modulate neutrophil apoptosis [115].

It is also interesting to mention that some AMPs have been identified to possess the potential to induce apoptosis via ROS accumulation especially, hydroxyl radicals giving rise to mitochondrial dysfunction. The mitochondria play a prominent role in the apoptotic pathway, during apoptotic induction by AMPs; cells undergo cytochrome c release, caspase activation, phosphatidylserine externalization, plasma or mitochondrial membrane depolarization, DNA and nuclei damage, cell shrinkage, apoptotic body formation, and membrane blebbing [111].

For instance, Lee and co-workers reported that coprisin a 43-amino acid defensin-like peptide from *C. tripartitus* induced apoptosis in *C. albicans* by inducing mitochondrial membrane potential dysfunction leading to cytochrome *c* release as well as activation of metacaspases via intracellular ROS and hydroxyl radical increase. They concluded that coprisin could be a model molecule for a large family of novel AMPs possessing apoptotic activities [70]. Collectively, it implies that AMPs could be explored in the treatment of HIDs because of their ability to resolve inflammatory processes and maintain balance in homeostasis either by inhibiting excessive apoptosis that could contribute in the pathogenesis of HIDs or induce apoptosis during inflammatory processes, thereby delaying or preventing the pathogenesis and progression of HIDs.

## 11. The Promise of AMP Therapy

In search for more efficient therapies with transient side effects in the treatment of HIDs, new research vistas is to screen and develop new therapies by exploring AMPs that are cytotoxic against multi-drug resistance (MDR) cells as well as possessing immunomodulatory activities. Even though the efficacy of these AMPs is yet to be tested in treating HIDs, the promise they hold is definitely worth mentioning. Numerous *in vitro* as well as animal studies strongly propose AMPs as suitable for developing novel therapeutic agents in combating various HIDs. However, the question of how these peptides will be delivered into human body as well as directing them to the right target organs in the treatment of various HIDs needs to be addressed. Consequently, future studies will focus on these and the regulatory mechanisms of AMPs in various HIDs.

In our own opinion, based on the aforementioned, we propose that AMPs will be an excellent candidate with great potential for the treatment and management of various HIDs (see model in Figure 1), firstly because of their immunomodulatory activities combined with their ability to induce angiogenesis and wound repair when necessary. Secondly, AMPs can prevent excessive ROS production by adequately mopping it up from biological systems, thereby maintaining balance between normal inflammatory response and excessive apoptosis.

Thirdly, due to their selective cytotoxic ability via lysis of the cell membrane and selective induction of intrinsic apoptotic pathway as well as their ability to inhibit angiogenesis, if need be. Lastly, because they possess the potential to selectively suppress key inflammatory signalling molecules such as NF-*κ*B, IL-1β and TNF-α. In conclusion, for the treatment and management of HIDs, a careful balance must be maintained between ROS production, inflammatory response and apoptosis. Therefore, AMPs are promising candidates in novel drug design due to their size and properties, diverse modes of actions associated with their broad spectrum of activities and evidence of reduced rate of resistance.
